# An individualized immune prognostic signature in lung adenocarcinoma

**DOI:** 10.1186/s12935-020-01237-4

**Published:** 2020-05-07

**Authors:** Liangdong Sun, Gening Jiang, Diego Gonzalez-Rivas, Peng Zhang

**Affiliations:** 1 Department of Thoracic Surgery, Shanghai Pulmonary Hospital, Tongji University School of Medicine, Shanghai, China; 2 Department of Thoracic Surgery, The First Affiliated Hospital of Wenzhou Medical University, Wenzhou, 325035 Zhejiang People’s Republic of China

**Keywords:** Lung adenocarcinoma, Immune risk score, Immune clinical score, Prognosis, Immunotherapy

## Abstract

**Background:**

Tumor immune infiltration is closely associated with clinical outcome in lung cancer. We aimed to develop an immune signature to improve the prognostic predictions of lung adenocarcinoma (LUAD).

**Methods:**

We applied “Cell type Identification by Estimating Relative Subsets of RNA Transcripts” method to quantify the fraction of 22 leukocyte cells from six public microarray datasets. Four datasets from GPL570 were treated as the training cohort and two datasets from GPL96 and GPL10379 as the validation cohorts. An immune risk score (IRS) based on leukocyte cell fraction was established by least absolute shrinkage and selection operator cox regression model.

**Results:**

IRS consisting of 6 types of leukocytes was constructed in the training dataset. In the training cohort (520 patients), the IRS stratified patients into high-IRS group (215 patients) and low-IRS group (305 patients) with significant differences in overall survival (OS) (HR: 2.77, 95% CI 2.08–3.06). Multivariate analysis including age, gender, stage, IRS and tumor purity revealed the IRS to be an independent prognostic factor in all datasets (training: HR: 10.71, 95% CI 5.72–20.07; validation-1: HR 2.68, 95% CI 1.15–6.27; validation-2: HR 3.71, 95% CI 1.33–10.33); all p < 0.05). IRS was significantly positively correlated to the expression levels of PD1, PDL1, CTLA and LAG3 (all p < 0.001). When integrated with clinical characteristics including stage and age, the composite immune and clinical signature presented with improved prognostic accuracy than IRS (mean C-index 0.66 vs. 0.60).

**Conclusions:**

The proposed immune-clinical signature could predict OS in patients with LUAD effectively.

## Background

Non-small cell lung cancer accounts for 85% of all lung cancers, the most common cancer and cause of cancer-related mortality world widely [1]. Lung adenocarcinoma (LUAD) is the most diagnosed histological subtype of non-small cell lung cancer [2, 3]. Due to the presence of metastatic disease at an early stage, the prognosis for patients with LUAD is generally poor, with average 5-year survival rates of < 20% [4]. Conventionally, clinical decisions regarding cancer treatment and prognosis are based primarily on the AJCC staging system [5].

However, increasing evidence has revealed the clinical importance of tumor-infiltrating immune cells in lung cancer [6–10], combining the survival impact of immune cells with the AJCC staging system could enable clinicians to predict patient survival outcomes more accurately. Therefore, understanding the immune components by gene expression-based algorithms may be helpful for promoting studies of immune response in LUAD. The availability of public genomic datasets provides an ideal resource for large-scale gene expression analysis to identify reliable lung cancer biomarkers [11].

High resolving power is a key benefit of “Cell type Identification by Estimating Relative Subsets of RNA Transcripts” (CIBERSORT), which applies LM22 signature matrix to quantify the relative proportions of 22 immune cell types [12]. Because of the superiority of CIBERSORT algorithm over other methods regarding noise, closely related cell subsets and unknown cell types, it has received increasing attention and has been successfully applied to quantify the composition of immune cells in colon, breast, liver cancer and LUAD [13–17].

Therefore, we used the estimated proportions of 22 leukocytes derived from microarray gene expression data to construct and validate an IRS for patients with LUAD. To combine the complementary value of IRS for overall survival (OS) with clinical characteristics, we integrated the IRS with clinical factors to develop a composite prognostic signature, which showed improved prediction of LUAD prognosis.

## Methods

### Datasets

The gene expression data and corresponding clinical characteristics of LUAD patients from Affymetrix^®^ (Affymetrix, Santa Clara, California, USA) were downloaded from the Gene Expression Omnibus (GEO) websites. Datasets selection criterion was as follows: 1) probe-level CEL files of microarray data were available; 2) the basic clinicopathological information (age, gender, stage and survival information) was available; 3) the sample size was larger than 180. Therefore, six datasets (GSE31210 [18], GSE30219 [19], GSE37745 [20], GSE50081 [21], GSE68465 [22] and GSE72094 [23]) were enrolled into our study. Four GEO datasets (GSE30219, GSE31210, GSE37745 and GSE50081) from GPL570 were treated as the training cohort. Moreover, we employed two independent GEO datasets, GSE68465 from GPL96 and GSE72094 from GPL10379, as the validation cohorts.

### Re-analysis of microarray data

Six GEO datasets were downloaded as probe-level CEL files. Then, the microarray data were normalized using Robust multiarray average (RMA) method with the affy and simpleaffy packages. The datasets used in the training cohort were quantile normalized after adjusting for batch effects using “combat” function (sva package, R 3.5.3) [24].

### Estimation of immune cell type fractions

Gene expression data were subsequently analyzed using the LM22 gene signature and CIBERSORT method to estimate the fractions of 22 tumor infiltrating leukocytes subsets [12]. The CIBERSORT algorithm is well developed and has been verified by fluorescence-activated cell sorting [12]. CIBERSORT derives a p value for the deconvolution of each sample using Monte Carlo sampling, providing a measure of confidence for the results. Patients with a CIBERSORT output of p < 0.05 indicated that the results of the estimated fractions of immune cell populations can be considered accurate [14]. For each tumor sample, the final CIBERSORT output estimates were normalized and the sum of all estimates of immune cell type fractions yields to one.

### Study population and clinical variables

Samples with CIBERSORT *p* value ≥ 0.05 were excluded, as were those with normal and non LUAD samples and patients for whom survival information or relevant clinical information was unknown. Clinical information including age, gender and TNM stage was collected. In this study, tumors were staged following the seventh edition of the AJCC staging system [25]. “Estimation of STromal and Immune cells in Malignant Tumours using Expression data” (ESTIMATE) algorithm was applied to calculate the stromal and immune scores of each sample and tumor purity can be evaluated using the formula reported before [26].

### Construction of IRS

The survminer package [27] was applied to determine the optimal cut-off values for each immune cell fraction in the training dataset. Then, the leukocyte fraction level was scored as 0 or 1; a leukocyte fraction level of 1 was assigned when the fraction of one type of leukocyte was more than the corresponding cut-off value; otherwise the fraction level was 0. Thus, the 22 leukocytes were then analyzed as binary variables. To minimize the risk of overfitting, a cox proportional hazards regression model combined with the least absolute shrinkage and selection operator (LASSO) [28] was applied to identify the most important prognostic immune cells, and the optimal values of the penalty parameter λ were determined by tenfold cross-validations at 1 SE beyond the minimum partial likelihood deviance in the training dataset [29]. An IRS model was constructed based on the selected immune cells using lasso cox regression coefficients derived from the training cohort. To separate patients into low- or high-IRS groups, the optimal IRS cutoff was also generated based on the association between IRS and OS using the survminer package.

### Validation of the IRS

The predictive value of IRS for OS was evaluated in all patients and in subgroups stratified by age, gender, TNM-stage and tumor purity in the training dataset, validation dataset-1 and validation dataset-2 with univariate cox analysis. We also combined IRS with other available variables in multivariate analysis (MVA).

### Establishing and validation of immune clinical score

According to the results of MVA in the training dataset, IRS, age and stage were significantly associated with OS. Thus, we integrated IRS, age, and stage to composite an immune clinical score (ICS) using cox proportional hazards regression in the training dataset. Stage was treated as continuous variable: stage I was assigned as 1; II, as 2; III, as 3; and IV, as 4. The prognostic performance of continuous ICS was compared with that of the IRS in terms of C-index. Meanwhile, the sensitivity and specificity of the OS prediction based on the IRS and ICS were evaluated using a time-dependent receiver operating characteristic (ROC) curve [30]. Similar to the aforementioned method for determining the optimal cutoff of IRS, the cutoff value for ICS was also generated using the survminer package. Restricted mean survival time (RMST) represents the life expectancy at 10 years for training dataset and validation dataset-1 and at 4 years for validation dataset-2 because of shorter follow-up time. The performance of binary IRS and ICS was evaluated in terms of the RMST ratio between low- and high-risk groups [31]. Accordingly, a higher RMST ratio indicates a larger prognostic difference.

### Statistical analysis

All statistical analysis was conducted using R software (version 3.5.3) and SPSS software (version 25.0). The correlations between the IRS and mRNA expression level of corresponding genes were analyzed using Pearson’s correlation test. Gene set enrichment analysis (GSEA) was used to identify the pathways that were significantly enriched in high-IRS and low-IRS groups [32]. Kaplan–Meier method was used to generate survival curves and significance of differences was compared using the log-rank test. Hazard ratios for univariate analysis were calculated using univariate cox proportional hazards regression model. The RMST ratio was estimated with survRM2 package [33]. All statistical tests were two-sided and P values less than 0.05 were considered statistically significant.

## Results

### Patient characteristics

**Fig. 1:**
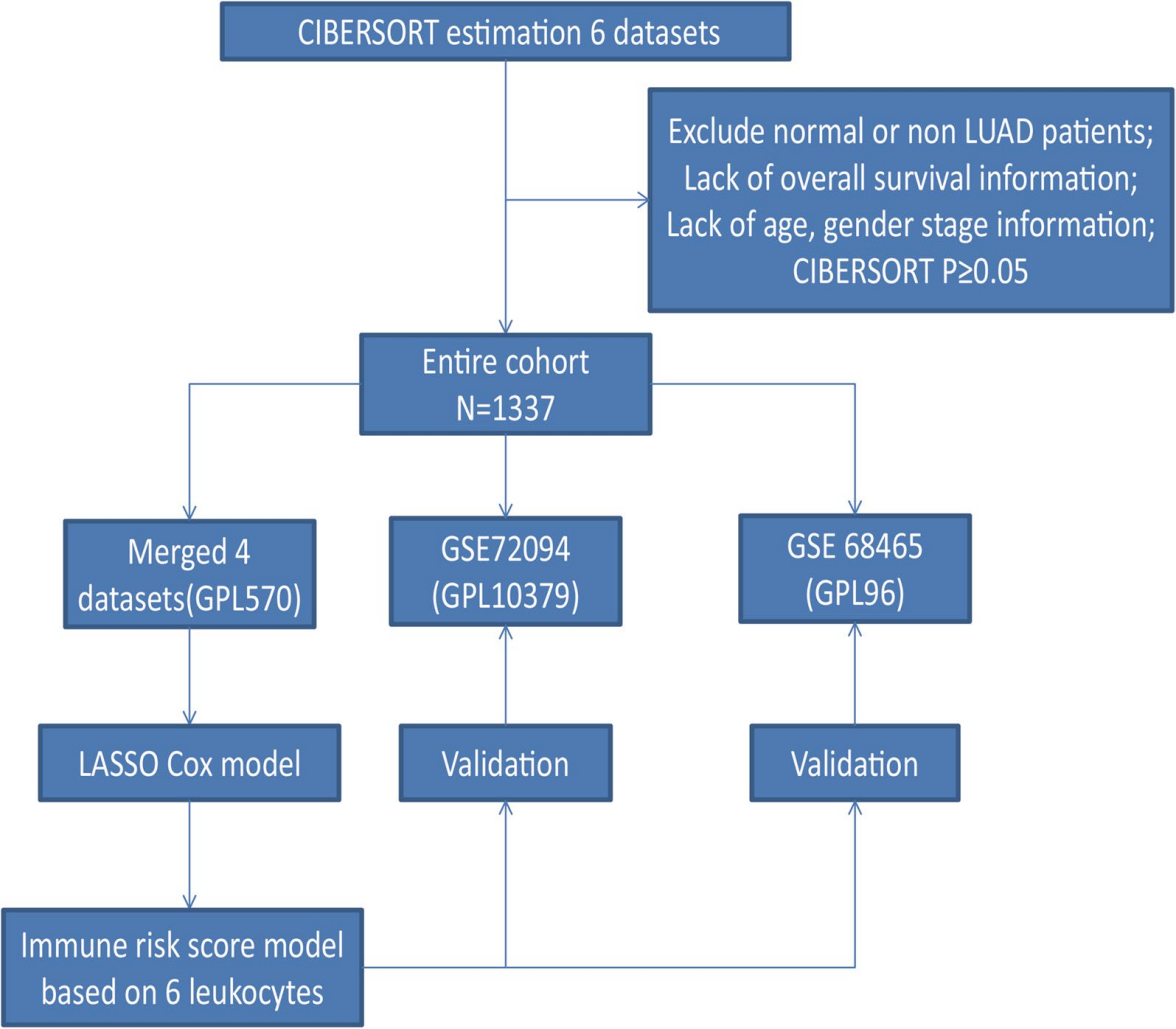
Flow chart of data collection and analysis. *CIBERSORT* Cell type Identification by Estimating Relative Subsets of RNA Transcripts, *LASSO* least absolute shrinkage and selection operator, *LUAD* lung adenocarcinoma

The patient selection criteria and workflow chart are shown in Fig. 1. After applying data filter scheme, 1337 LUADs were used for further analysis. The mean age at diagnosis was 65.20 years and 657 (49.14%) patients were male. Most patients (88.33%) were early stage (stage I or II) diseases and the mean tumor purity was 0.50. Detailed patient characteristics are listed in Table 1.

**Table 1:** Patients’ basic characteristics

	No. of patients (n = 1337)
Affymetrix^®^ platform	
GPL570	520 (38.89%)
GPL96	436 (32.61%)
GPL10379	381 (28.50%)
Age (years),mean(sd)	65.20 (10.01)
Sex ratio (M: F)	657:680
Stage	
I	753 (56.32%)
II	428 (32.01%)
III	138 (10.32%)
IV	18 (1.35%)
Purity, mean (SD)	0.50 (0.15)

### Derivation of the IRS

**Fig. 2:**
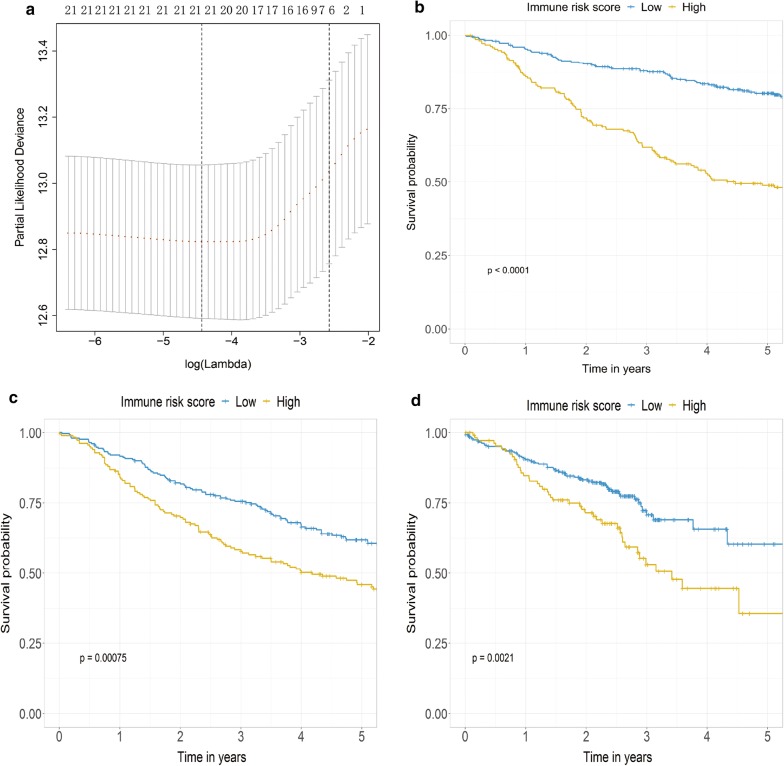
IRS construction and validation. **a** Partial likelihood deviance of different numbers of variables revealed by the LASSO regression model. The red dots represent the partial likelihood deviance values, the grey lines represent the standard error (SE), the two vertical dotted lines on the left and right, respectively, represent optimal values by minimum criteria and 1-SE criteria. **b**–**d** Kaplan–Meier curves of OS between high and low IRS groups in the training cohort (**b**), validation dataset-1 (**c**) and validation dataset-2 (**d**); *IRS* immune risk score, *LASSO* least absolute shrinkage and selection operator, *OS* overall survival

The optimal cut-off values were generated for 22 leukocytes in the training cohort (Additional file 1: Table S1). LASSO cox regression analysis was used to build an IRS model (Fig. 2a). Six leukocyte subsets were identified to calculate the IRS as following:

$$\begin{aligned} {\text{IRS}} & = \left( {\left( { - 0.16527275} \right) \times {\text{fraction}}\;{\text{level}}\;{\text{of}}\;{\text{plasma}}\;{\text{cells}}} \right) + \left( {\left( { - 0.294722231} \right) \times {\text{fraction}}\;{\text{of}}\;{\text{T}}\;{\text{cells}}\;{\text{CD}}4\;{\text{Memory}}\;{\text{resting}}} \right) \\ & \quad + \left( {\left( {0.5352580} \right) \times {\text{fraction}}\;{\text{level}}\;{\text{of}}\;{\text{Macrophages}}\;{\text{M}}0} \right) + \left( {\left( { - 0.3803774} \right) \times {\text{fraction}}\;{\text{level}}\;{\text{of}}\;{\text{Mast}}\;{\text{cells}}\;{\text{resting}}} \right) \\ & \quad + \left( {\left( {0.21355828} \right) \times {\text{fraction}}\;{\text{level}}\;{\text{of}}\;{\text{Mast}}\;{\text{cells}}\;{\text{activated}}} \right) + \left( {\left( {0.3492314} \right) \times {\text{fraction}}\;{\text{level}}\;{\text{of}}\;{\text{Neutrphils}}} \right) \\ \end{aligned}.$$

Patients in the training cohort were then assigned into a high- IRS group (215 patients) and low-IRS group (305 patients) by the cut-off value (− 0.1652727). The Kaplan–Meier curve showed the patients in the high-IRS group presented with a significantly worse OS in the training dataset (HR 2.77, 95% CI 2.78–3.67, p < 0.01) (Fig. 2b). The median OS was 11.19 years in low-IRS group vs. 4.47 years in the high-IRS group (p < 0.01). The association between the IRS and OS was further investigated in the multivariable Cox regression model (HR: 10.71, 95% CI 5.72–20.07) (Table 2).

**Table 2:** Multivariate cox analysis of immune risk score and clinical variables in training dataset, validation dataset-1 and validation dataset-2

	Training dataset HR (95% CI)	p value	Validation dataset-1 HR (95% CI)	p value	Validation dataset-2 HR (95% CI)	p value
IRS	10.71 (5.72–20.07)	< 0.01	2.68 (1.15–6.27)	0.02	3.71 (1.33–10.33)	0.01
Age	1.03 (1.02–1.05)	< 0.01	1.03 (1.01–1.04)	< 0.01	1.00 (0.98–1.03)	0.65
Gender male	1.08 (0.81–1.44)	0.60	1.22 (0.93–1.59)	0.14	1.69 (1.14–2.51)	< 0.01
Stage						
I	Reference					
II	2.12 (1.56–2.89)	< 0.01	1.85 (1.28–2.69)	< 0.01	2.13 (1.30–3.51)	< 0.01
III	2.52 (1.38–4.59)	< 0.01	5.57 (3.66–8.48)	< 0.01	3.37 (2.05–5.54)	< 0.01
IV	2.58 (0.82–8.18)	0.11	NA	NA	4.02 (1.78–9.08)	< 0.01
Purity	1.31 (0.48–3.53)	0.60	1.41 (0.52–3.85)	0.50	0.95 (0.23–3.91)	0.95

*IRS* immune risk score, *CI* confidence interval, *HR* hazard ratio

### Validation of IRS for predicting overall survival in the validation dataset-1 and validation dataset-2

To ensure that the constructed IRS possessed predictive value for OS in different cohorts, the same formula derived from the training cohort was applied to the validation dataset-1 and validation dataset-2. Patients were assigned to high- or low-IRS group by the cut-off values acquired from the corresponding cohort (validation dataset-1, 0.04828553; validation dataset-2, − 0.03803774). In the validation dataset-1, 183 patients were assigned into low-IRS group and 253 patients were assigned into high-IRS group. As for validation dataset-2, 109 patients were assigned into low-IRS group and 272 patients were assigned into high-IRS group. Consistent with the findings in the training cohort, patients in the high-IRS group presented with a significantly worse OS than those in the low-IRS group in the validation dataset-1 (HR 1.56, 95% CI 1.20–2.02) (Fig. 2c) and the validation dataset-2 (HR 1.83, 95% CI 1.24–2.78) (Fig. 2d). The median OS was 7.81 years in low-IRS group vs. 6.16 years in the high-IRS group in the validation dataset-1 and 4.30 years in low-IRS group vs. 3.48 years in the high-IRS group in the validation dataset-2 (both p < 0.01). The IRS remained as an independent prognostic factor in MVA, after adjusting for clinical characteristics such as age, gender, TNM stage and purity in validation dataset-1 (HR 2.68, 95% CI 1.15–6.27) and the validation dataset-2 (HR 3.71, 95% CI 1.33–10.33) (both p < 0.05) (Table 2).

### The IRS was associated with OS in early stage patients

To further investigate the impacts of clinical characteristics on the prognostic values of the IRS, we conducted stratified analysis according to the baseline characteristics. As shown in Table 3, LUADs were stratified by available baseline characteristics (including age, gender, TNM stage and tumor purity). According to the results of stratified analysis, the IRS discriminated patients with early-stage (I and II) LUAD into significantly different prognostic groups in training dataset, validation dataset-1 and validation dataset-2 (all p < 0.01) (Table 3). When considering LUADs with stage I disease only, the IRS remained highly prognostic for the meta-overall dataset (combined HR: 2.01, 95% CI 1.26–3.22; p < 0.01) (Additional file 1: Fig. S1).

**Table 3:** The association between high- and low- immune risk score and OS of LUAD patients in training, validation dataset-1 and validation dataset-2

	Training dataset (N = 520)			Validation dataset-1 (N = 436)			Validation dataset-2 (N = 381)		
	(High/low)	HR (95% CI)	p value	(High/low)	HR (95% CI)	p value	(High/low)	HR (95% CI)	p value
Total	215/305	2.77 (2.08–3.67)	< 0.01	183/253	1.56 (1.20–2.02)	< 0.01	109/272	1.83 (1.24–2.78)	< 0.01
Age									
< 70	165/236	2.97 (2.09–4.24)	< 0.01	119/171	1.53 (1.11–2.13)	0.01	56/119	1.93 (1.06–3.52)	0.03
≥ 70	50/69	2.87 (1.76–4.69)	< 0.01	64/82	1.71 (1.12–2.63)	0.05	53/153	1.82 (1.08–3.05)	0.02
Gender									
Male	132/139	2.28 (1.57–3.31)	< 0.01	102/116	1.42 (1.01–2.00)	0.04	54/114	1.95 (1.14–3.33)	< 0.01
Female	83/166	3.47 (2.23–5.39)	< 0.01	81/176	1.66 (1.11–2.47)	0.01	55/158	1.66 (0.93–2.95)	0.09
Stage									
I/II	205/297	2.73 (2.03–3.65)	< 0.01	150/218	1.66 (1.23–2.25)	< 0.01	85/226	2.06 (1.29–3.29)	< 0.01
III/IV	10/8	2.71 (0.83–8.87)	0.10	35/33	0.95 (0.57–1.59)	0.85	24/46	1.20 (0.59–2.47)	0.61
Purity									
< 0.5	146/174	3.33 (2.30–4.82)	< 0.01	52/61	1.95 (1.11–3.41)	0.02	42/84	1.70 (0.89–3.24)	0.11
≥ 0.5	69/131	2.11 (1.33–3.33)	0.01	131/192	1.50 (1.11–2.02)	< 0.01	67/188	1.93 (1.17–3.13)	< 0.01

*CI* confidence interval, *HR* hazard ratio, *LUAD* lung adenocarcinoma, *OS* overall survival

### Biological phenotypes associated with the IRS model

**Fig. 3:**
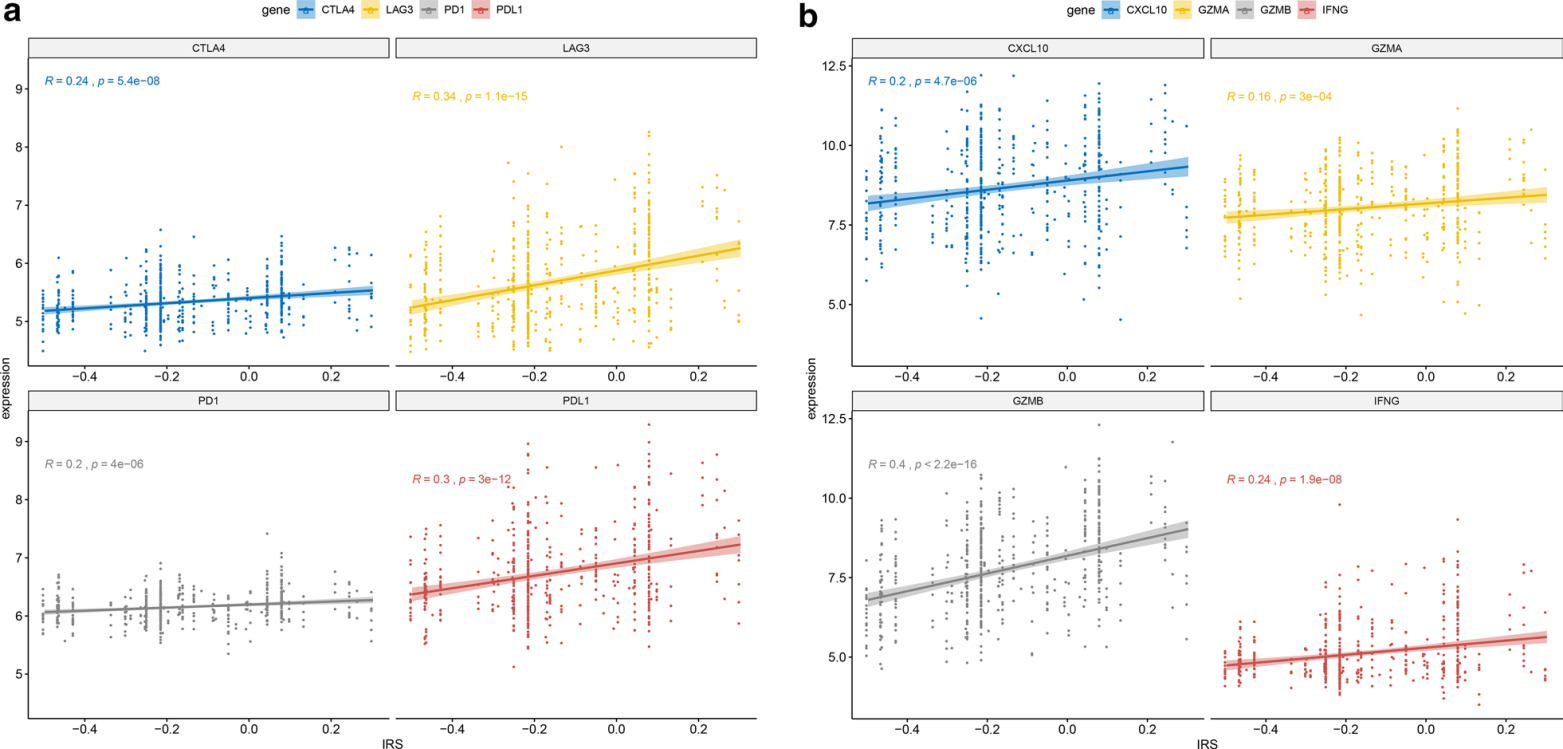

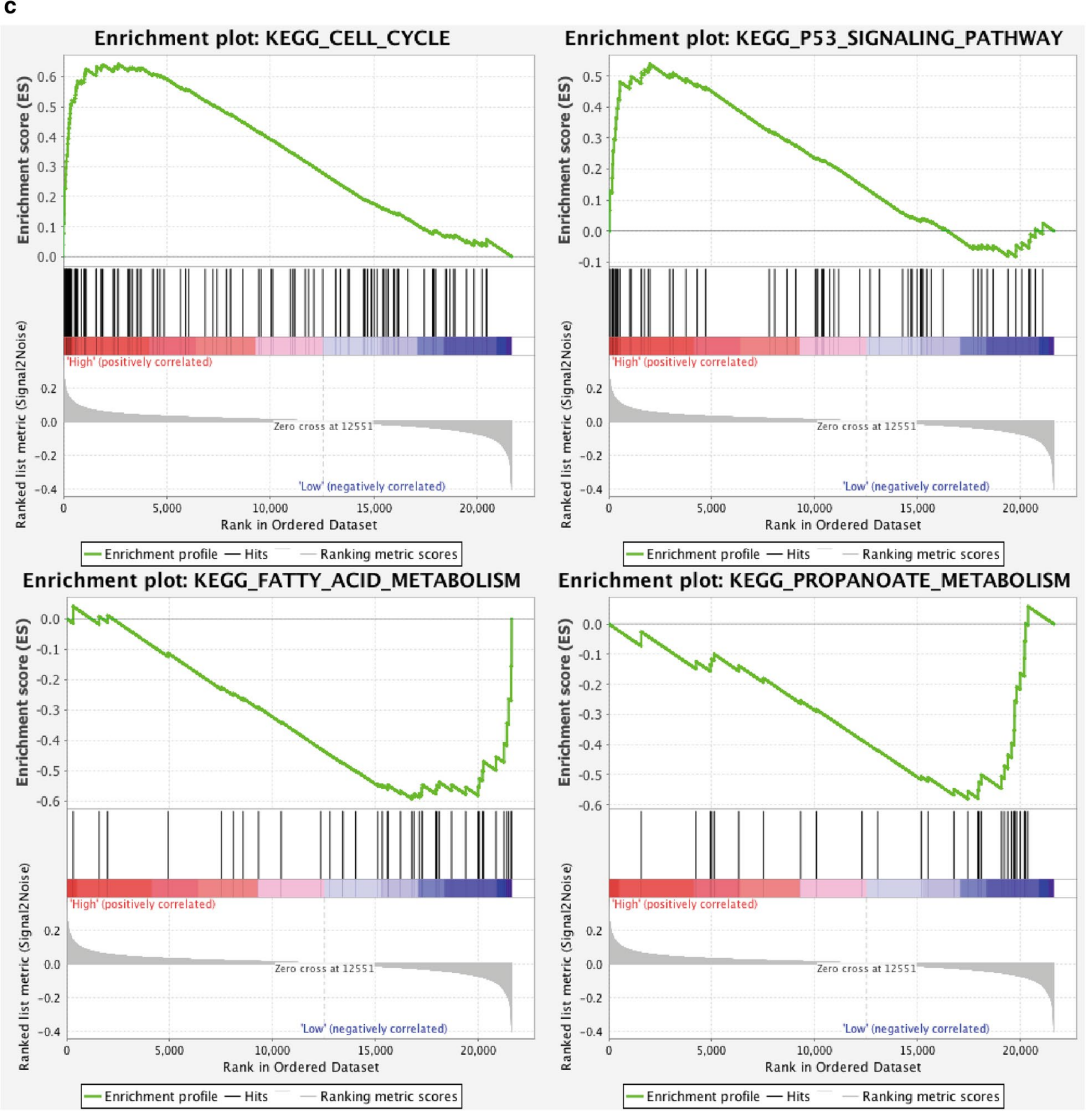
Biological function of IRS in the training dataset. **a** The correlation between IRS and immune checkpoint regulators and y axis represents the expression levels of certain genes. **b** The correlation between IRS and immune-activated related transcripts and y axis represents the expression levels of certain genes. **c** Gene set enrichment analysis delineates biological pathways between high- and low-IRS groups. *IRS* immune risk score

Gene expression data were analyzed to investigate the potential biological phenotypes associated with the IRS model in the training dataset. Firstly, we specially focused on some immune check points and the correlation plot depicted in Fig. 3a showed that the IRS was significantly positively correlated to the expression levels of PD1, PDL1, CTLA and LAG3 (all p < 0.001). Secondly, as for some immune-activated related transcripts such as GZMA, GZMB, CXCL10 and IFNG, IRS was also significantly positively correlated to the expression levels of them (all p < 0.001) (Fig. 3b).Finally, we performed GSEA to illuminate the biological functions of the IRS model. The results showed that in the high-IRS group genes were significantly enriched in multiple biological processes such as cell cycle pathway and p53 signaling pathway, while in the low-IRS group genes were associated with the metabolism-related gene set, including fatty acid metabolism and propanoate metabolism (Fig. 3c).

### Integrated prognostic score combining the IRS with clinical factors

**Fig. 4:**
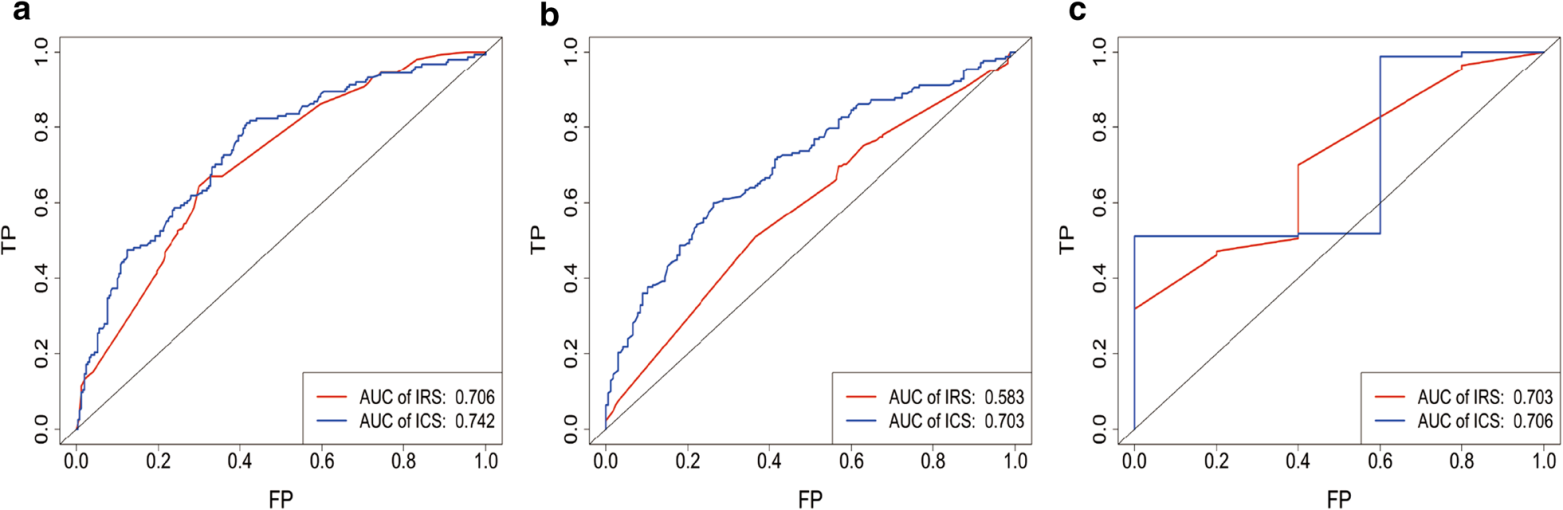
IRS and ICS measured by time-dependent ROC curves at 5 years in the training dataset (**a**), validation dataset-1 (**b**), and validation dataset-2 (**c**). *AUC* area under the curve, *IRS* immune risk score, *ICS* immune clinical score, *ROC* receiver operator characteristic

**Fig. 5:**
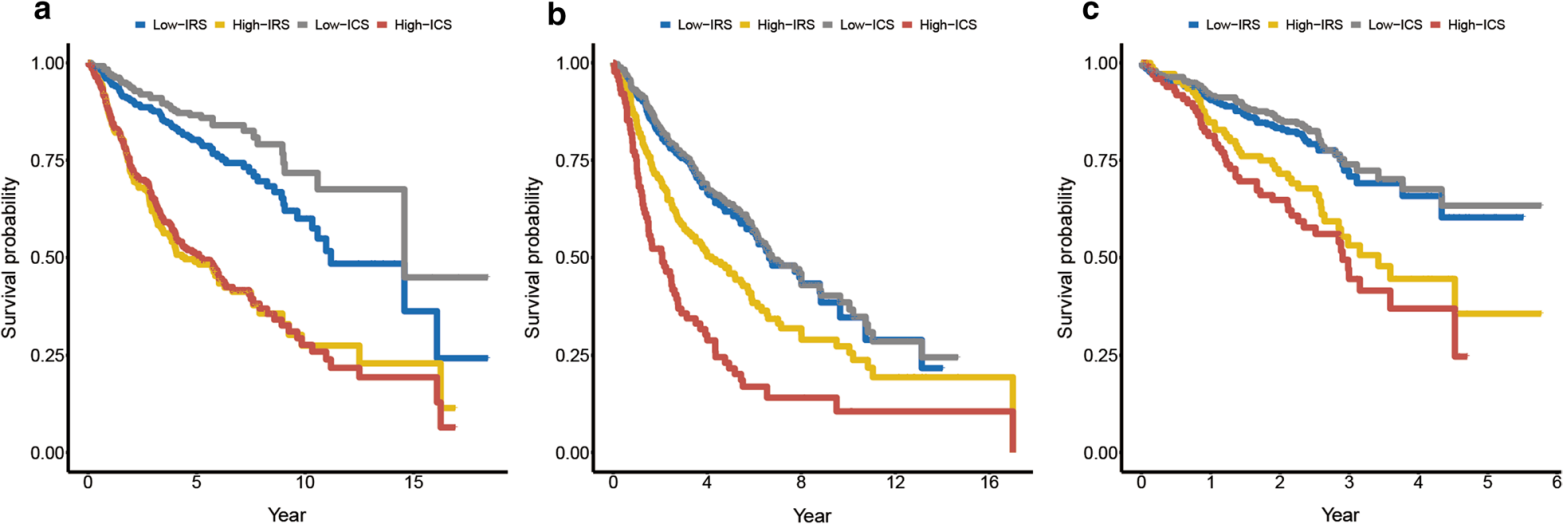
Kaplan–Meier curves for overall survival of all patients stratified by the IRS and the ICS in the training dataset (**a**), validation dataset-1 (**b**) and validation dataset-2 (**c**). *IRS* immune risk score, *ICS* immune clinical score

In MVA (Table 2), IRS, age and stage were prognostic factors in at least two datasets, implying their complementary value for predicting OS. To further improve prediction accuracy, we combined IRS, age and stage to fit a Cox proportional hazards regression model in the training cohort and derived an Immune Clinical Score (ICS): ${\text{ICS}} = \left( {2.68575 \times {\text{IRS}}} \right) + \left( {0.03221 \times {\text{age}}} \right) + \left( {0.50289 \times {\text{stage}}} \right)$. Improved estimation of OS was achieved by the continuous form of ICS compared with IRS (C-index, 0.66 vs. 0.64 in the training dataset) (Additional file 1: Table S2). The prognostic accuracy of the ICS as a continuous variable was also evaluated by time-dependent ROC analysis (Fig. 4a). An optimal cutoff of 2.135404 for stratifying patients was determined in the training dataset. Similar results were observed in binary form of the ICS compared with the IRS (RMST ratio, 1.56 vs. 1.47 in the training dataset) (Table 4 and Fig. 5a).

**Table 4:** RMST ratio between low- and high-risk groups based on immune risk score or immune clinical score in training, validation dataset-1 and validation dataset-2

RMST (years)	Immune risk score			Immune clinical score		
	Low risk (95% CI)	High risk (95% CI)	Ratio (95% CI)	Low risk (95% CI)	High risk (95% CI)	Ratio (95% CI)
Training dataset	8.01 (7.62–8.40)	5.45 (4.91–5.99)	1.47 (1.32–1.64)	8.66 (8.27–9.04)	5.55 (5.09–6.02)	1.56 (1.42–1.71)
Validation dataset-1	6.34 (5.87–6.83)	5.05 (4.49–5.62)	1.26 (1.10–1.44)	6.46 (6.06–6.87)	3.25 (2.56–3.93)	1.99 (1.60–2.48)
Validation dataset-2	3.29 (3.13–3.45)	2.85 (2.59–3.11)	1.16 (1.04–1.28)	3.36 (3.22–3.51)	2.60 (2.32–2.90)	1.29 (1.15–1.46)

*CI* confidence interval, *RMST* restricted mean survival time

## Discussion

In this study, we developed an immune prognostic signature based on the 6 leukocytes and validated it in two independent datasets from different platforms. The results showed a significantly discriminative ability of OS between patients with high- and low- IRS. In addition, IRS can further stratify clinically defined groups of patients (especially early-stage) into subgroups with different survival outcomes. The IRS was significantly positively correlated to the expression levels of some immune check points and immune-activated related transcripts. We further investigated the complementary value of IRS and clinical characteristics and found that integrating both could give a more accurate estimation of OS for patients with LUAD.

In recent years, immune profiling studies have taken up a research focus in cancer study [9]. In LUAD, several studies have explored the association between tumor-infiltrating lymphocytes and patients’ survival. High CD4^+^ T cell in stroma correlated with longer OS [34] and disease-specific survival [35] and plasma cell infiltration was related to worse prognosis in LUAD patients [36]. It has been argued that macrophages may have a potential role in lung cancer by supporting both host-defense and tumor progression [37]. Mast cells were regarded a double-edged sword in cancer immunity: a higher density of mast cells was reported to correlate with improved survival in patients with LUAD [38] but activated mast cells presented with potential to exert immunosuppressive effects [39] and Takanami et al. [40] found increased mast cell infiltration in LUAD was associated with worse prognosis. Neutrophils represented a significant portion of infiltrating inflammatory cells and high neutrophil density was associated with a higher risk of relapse [41] and was a negative prognostic factor in LUAD [42]. To explore the potential role of tumor-infiltrating lymphocytes may require investigation comprehensively in tumor microenvironment.

Several models [43–45] based on immune cells have already been reported to present with strong ability for predicting prognosis in various types of tumors. Immunohistochemistry (IHC) is an important means of investigating tumor immune micro-environment [46] in these studies. But IHC suffers from limitations in available phenotypic markers [47] and provides only a snap shot of the tumor IME assayed on the slide [17]. In addition, a standardized measurement criterion of the intensity of protein staining, and subsequently quantitation of protein expression, was also difficult for IHC in nature [48].

As an alternative, continuously accumulating public genomic data provided an ideal resource for large-scale analysis of the immune landscape, and multiple computer-based algorithms have already been developed to perform such analysis [49]. The candidate immune cells used to construct the IRS were quantified based on a high-throughput gene expression data using bioinformatics tool CIBERSORT. By applying this computational method to public genomic data, it was possible to overcome some technical limitations of IHC and give an expanded insight into the immune profile in tumor. With further use of LASSO Cox regression model, functioning as a statistical method for screening prognosis-related immune cells to construct the IRS model, the predictive ability could be enhanced significantly [50–52]. We compared our results with reports from Yang et al. and found that our IRS model using much less immune cell types showed better predictive ability than IRS model from Yang [53] (mean 5-year AUC of 0.66 vs. 0.62). In addition, we included tumor purity into our analysis to adjust IRS, enabling our results more reliable. The value of the IRS was confirmed in two non-overlapping validation cohorts, indicating its excellent reproducibility for LUAD.

Patients with early-stage lung cancer are also at substantial risk for recurrence and death [54], even after complete surgical treatment and the use of adjuvant therapy in early-stage lung cancer remains controversial. An important finding in our analysis was that the IRS was significantly associated with OS in stage I/II LUAD patients. The prognostic role of IRS was also confirmed in patients with stage I for meta-overall dataset, implying IRS may provide a powerful prognostic indicator for selecting potential patients benefiting from additional therapy.

FDA has approved IHC PDL1 expression as predictive biomarker for response to anti-PD1 therapy for patients with NSCLC [55, 56]. Since our study revealed obvious enrichment of multiple immune checkpoint markers and immune activation transcripts, especially PDL1, in the high-IRS group, it is reasonable to speculate that immunotherapy might also be a preferable choice for patients in this group. Although patients in high-IRS group presented with poor OS, the application of immunotherapy may bring potential survival benefit. Further studies are warranted to explore whether the IRS model can predict the response of patients with LUAD to immunotherapy. The ICS integrating the IRS and baseline characteristics not only helped clinicians predict patient outcomes more precisely but boosted its procedure for translation into clinic utility.

This study has some limitations. First, as all patients in this study were selected retrospectively, the potential bias relating to unbalanced clinical features with treatment heterogeneity cannot be avoided. Secondly, the gene expression profiles used here were all derived from a core sample of tumor tissue, making it impossible for the location of the immune cell to be taken into consideration when establishing the prognostic IRS model. Thirdly, only signatures validated in independent cohorts of patients with full clinical annotation available could be applied clinically, and thus further investigations should focus on clinical validation for IRS, which may provide more evidence for its translation into clinical practice.

## Conclusion

In conclusion, our study demonstrates the utility of consideration of tumor infiltrating leukocytes in the prognosis prediction of LUAD and may provide additional information and strategies for immunotherapy. Prospective studies are needed to further test its analytical accuracy for estimating prognosis and to validate its clinical utility in individualized management of LUAD patients.

## Supplementary information

**Additional file 1.** An individualized immune prognostic signature in lung adenocarcinoma. Liangdong Sun, Gening Jiang, Diego Gonzalez-Rivas and Peng Zhang.

## Data Availability

Six GEO datasets (GSE31210, GSE30219, GSE37745, GSE50081, GSE68465 and GSE72094) used in this study could be downloaded from GEO database (https://www.ncbi.nlm.nih.gov/geo/).

## Abbreviations

LUAD: Lung adenocarcinoma
CIBERSORT: Cell type Identification by Estimating Relative Subsets of RNA Transcripts
OS: Overall survival
IRS: Immune risk score
GEO: Gene Expression Omnibus
RMA: Robust multiarray average
ESTIMATE: Estimation of STromal and Immune cells in Malignant Tumours using Expression data
LASSO: Least absolute shrinkage and selection operator
MVA: Multivariate analysis
ICS: Immune clinical score
ROC: Receiver operating characteristic
RMST: Restricted mean survival time
GSEA: Gene set enrichment analysis
IHC: Immunohistochemistry
