# Supplementary material for: An individualized immune prognostic signature in lung adenocarcinoma
Source: Cancer Cell Int. 2020 May 7;20:156. doi: 10.1186/s12935-020-01237-4 (PMC7206750; doi:10.1186/s12935-020-01237-4)
Supplement: Supplementary file 1 — Additional file 1. An individualized immune prognostic signature in lung adenocarcinoma. Liangdong Sun, Gening Jiang, Diego Gonzalez-Rivas and Peng Zhang. [file 12935_2020_1237_MOESM1_ESM.docx]

**An individualized immune prognostic signature in lung adenocarcinoma**

Liangdong Sun, Gening Jiang, Diego Gonzalez-Rivas and Peng Zhang

**Table S1 Cut-off value for 22 leukocytes**

| Immune cell types | Cut-off value |
| --- | --- |
| B cells naive | 0.032846381 |
| B cells memory | 0.021289977 |
| Plasma cells | 0.050385119 |
| T cells CD8 | 0.013742258 |
| T cells CD4^+^ naive | 0.001814741 |
| CD4^+^T cells memory resting | 0.035630328 |
| CD4^+^ T cells memory activated | 0.045729056 |
| T cells follicular helper | 0.041709256 |
| T cells regulatory | 0.005331567 |
| γδT cells | 0.073641037 |
| NK cells resting | 0.002500538 |
| NK cells activated | 0.022403042 |
| Monocytes | 0.030873465 |
| Macrophages M0 | 0.270816237 |
| Macrophages M1 | 0.139161422 |
| Macrophages M2 | 0.082319870 |
| Dendritic cells resting | 0.029725714 |
| Dendritic cells activated | 0.037005275 |
| Mast cells resting | 0.070601365 |
| Mast cells activated | 0.003474858 |
| Eosinophils | 0.002879480 |
| Neutrophils | 0.006620389 |

NK: natural killing

**Table S2** The C-index for the immune risk score and immune clinical score in train, validation dataset-1 and validation dataset-2

| Datasets | Immune risk score | Immune clinical score |
| --- | --- | --- |
| Training dataset | 0.64 | 0.66 |
| Validation dataset-1 | 0.58 | 0.66 |
| Validation dataset-2 | 0.57 | 0.65 |

**Fig S1: Meta-analysis of the prognostic value of immune risk score in lung adenocarcinoma with stage I**

**
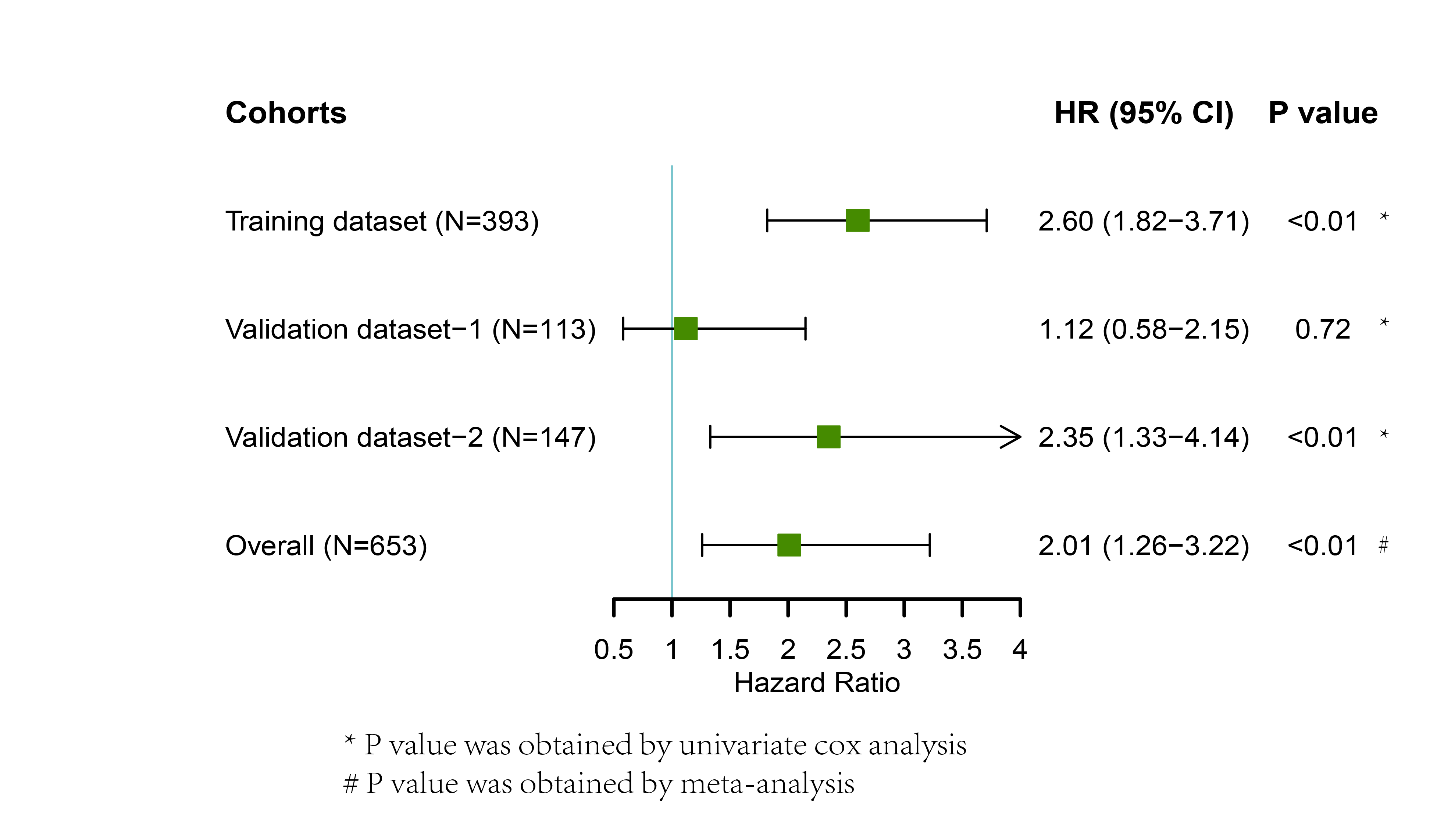
**
